# An Electromagnetic Sensor for the Autonomous Running of Visually Impaired and Blind Athletes (Part I: The Fixed Infrastructure)

**DOI:** 10.3390/s17020364

**Published:** 2017-02-14

**Authors:** Marco Pieralisi, Valentina Di Mattia, Valerio Petrini, Alfredo De Leo, Giovanni Manfredi, Paola Russo, Lorenzo Scalise, Graziano Cerri

**Affiliations:** 1 Department of Information Engineering, Marche Polytechnic University, 60131 Ancona, Italy; marco.pieralisi@univpm.it (M.P.); v.petrini@univpm.it (V.P.); a.deleo@univpm.it (A.D.L.); g.manfredi@univpm.it (G.M.); paola.russo@univpm.it (P.R.); g.cerri@univpm.it (G.C.); 2 Department of Industrial Engineering and Mathematical Science, Marche Polytechnic University, 60131 Ancona, Italy; l.scalise@univpm.it

**Keywords:** electromagnetic travel aids, visually impaired athletes, electromagnetic safety

## Abstract

Sport is one of the best ways to promote the social integration of people affected by physical disability, because it helps them to increase their self-esteem by facing difficulties and overcoming their disabilities. Nowadays, a large number of sports can be easily played by visually impaired and blind athletes without any special supports, but, there are some disciplines that require the presence of a sighted guide. In this work, the attention will be focused on marathons, during which athletes with visual disorders have to be linked to the sighted guide by means of a non-stretchable elbow tether, with an evident reduction of their performance and autonomy. In this context, this paper presents a fixed electromagnetic infrastructure to equip a standard running racetrack in order to help a blind athlete to safely run without the presence of a sighted guide. The athlete runs inside an invisible hallway, just wearing a light and a comfortable sensor unit. The patented system has been homemade, designed, realized and finally tested by a blind Paralympic marathon champion with encouraging results and interesting suggestions for technical improvements. In this paper (Part I), the transmitting unit, whose main task is to generate the two magnetic fields that delimit the safe hallway, is presented and discussed.

## 1. Introduction

It is commonly known that a high level of fitness is associated with physical, social and mental well-being because it has a positive influence on many physical and psychological aspects of human life. This is most true when speaking about people affected by visual disabilities, because physical activity, in general, promotes their social integration by helping them to strengthen their self-esteem, facing difficulties and overcoming their disabilities. On the contrary, a sedentary life can lead to negative feelings of anxiety, low self-esteem, depression, low confidence and poor self-efficacy [1], exposing blind and visually impaired subjects to more health risks than sighted people. For example, a study published in 2007 [2] identified low levels of physical activity as an indirect cause of increased risk of stroke, osteoporosis, depression, hypertension, heart disease, diabetes and falls in blind people. Moreover, a relationship was found between sedentary lifestyle and decreased life satisfaction among the blind and the visually impaired.

In this context, many sports were specifically developed for people affected by visual impairments in order to promote their participation in a physical activity. Some examples are Torball and Goalball, which are team sports of three players per team, involving the use of balls with holes where noise bells are located to facilitate blind athletes [3].

There is also a large number of sports that, although not uniquely developed for visually impaired and blind athletes, can be easily played even by these subjects, because they do not require any special aids at all or just some simple supports. Judo and other athletics disciplines, such as jumping events, are examples of sports that require just an acoustic warning to help with orientation inside the Tatami or the take-off areas. No particular difficulties obstruct visually impaired people from swimming, because the unique special precaution is that a coach touches the blind subject's head with a stick to warn him about the end of the pool.

Conversely, some issues exist related to running. In fact, both professional and amateur runners need to be assisted by a sighted conductor able to guide the subject along the desired path. The need is so compelling that, during official races, the athlete has to wear particular glasses and has to be linked to a sighted runner, the “guide”, by means of a not stretchable elbow tether such that the distance between them never exceeds 50 cm [3]. This restriction can have important consequences on the athlete’s performance and autonomy.

Significant efforts to remove the important barriers in everyday life have been made in designing smart Electronic Travel Aids able to assist the visually impaired during walking in indoor and outdoor unknown environments [4]. Most of these devices are based on ultrasonic and optic sensors, but recent studies have demonstrated the possibility of using the electromagnetic (EM) technology to realize an efficient obstacle detection system, whose potential advantages with respect to the more traditional technologies have been investigated [5].

On the contrary, there is a lack of smart assistive devices specifically designed to improve the independence of visually impaired runners. A few examples are described in literature [6,7] but none of them are actually used or have been commercialized yet.

The idea of facing a problem not yet satisfactorily solved and the interesting results and potentialities shown by the EM travel aid [8] led the authors to set up the realization of low-cost EM systems able to improve the autonomy of visually impaired and blind people even during daily running.

The first idea we worked on was the realization of a system able to confine the blind runner inside a secure invisible hallway [9]. To this end, we equipped a mobile unit (it can be a vehicle or a bike) with two antennas generating two lateral narrow “EM walls”. Basically, when the mobile unit goes forward, the runner may safely follow it, simply paying attention to the sensors around his arms. Such sensors generate vibro-tactile warnings each time he is getting close to one of the borderlines, so as to encourage him to move back toward the central position where no more warnings are produced. The system has great flexibility and thus is suitable for any path, but, at the same time, it still requires the presence of a person, who drives the vehicle that precedes the blind athlete.

**Figure 1:**
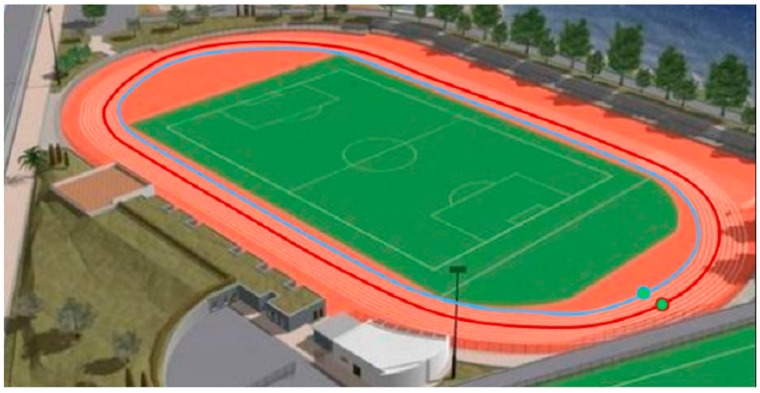
Example of system fixed infrastructure positioning in a stadium. Two concentric loops have the size of a standard athletic lane. Each loop is fed with different currents to identify the left and right side.

In this paper, we present an EM system developed and realized to allow a person with visual impairments to run along a racetrack completely free, without the need for a sighted guide. As with the system described above, a safe path is defined to let the athlete run autonomously, simply wearing a receiving device and following the vibro-tactile warnings generated by two sensors on his arms. The main difference with the system proposed in [9] is the employment of wires lying on the ground to realize the fixed infrastructure to be placed along the athletics track of a stadium. In detail, this solution relies on the generation of two EM boundaries with the use of two wires placed directly on the ground perimeter of a standard athletics track (400 m length), as schematically depicted in Figure 1. A variable current flows through each wire whose magnetic field can be detected by a coil (the receiver) worn by the runner. Therefore, the transmitter is made of two concentric giant rings and the receiver is a loop realized with a 40-turns flat cable wire able to increase its sensitivity.

The two parts composing the complete system—the transmitting and the receiving subunits—have been firstly theoretically studied, then realized and finally tested thanks to the collaboration of a blind volunteer. In particular, in this paper (Part I), the design and realization of the transmitting subsystem are reported while in a second related paper (Part II) the receiving subunit is described in detail together with some interesting tests with the blind runner.

The paper is divided as follows: Section 2 gives an overview of the system operating principle by means of an approximated theoretical model. Section 3 describes the design of the transmitting unit, focusing the attention on the evaluation of the impedance of the two wires lying on the ground and on the design of a suitable signal generator. A final discussion and some conclusions are reported in Section 4.

## 2. System Overview: An Approximated Theoretical Model

**Figure 2:**
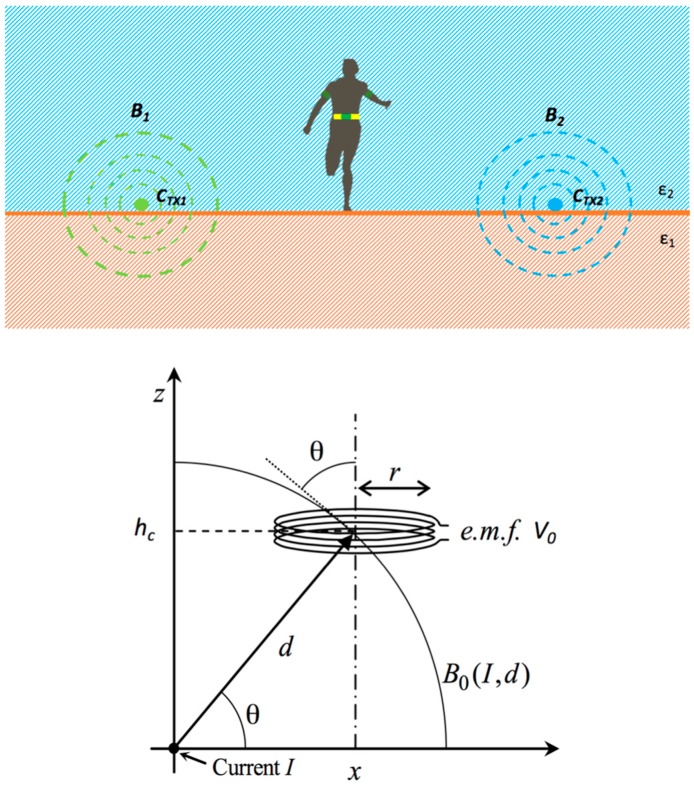
Top: Athlete between two wires emitting the electromagnetic signal, transverse section. Bottom: schematic representation of the geometrical parameters relative to the interaction between the magnetic field from a cable and the magnetic loop worn by the athlete.

In this section, the system operating principle, schematically represented in Figure 2, is sketched using a simple approximated model.

After positioning two wires on the ground and connecting them to the generator, a blind athlete will be able to run safely inside an invisible lane, following the vibration signals sent to his arms when he is getting too close to any of the boundaries.

The transmitting unit is composed by a radio frequency (RF) generator and two wires, C_TX1_ and C_TX2_, (length of about 400 m and cross section of 2.5 mm^2^) lying on the ground. Currents I_1_ and I_2_ flow along the wires and generate two induction magnetic fields—*B*_1_ and *B*_2_ respectively—as depicted in Figure 2 (top). The receiving unit is composed of a flat cable of *N* wires and a small circuit, which detects the two different electromotive forces (*e.m.f.*) induced by the two magnetic fields and then it evaluates the position of the runner inside the lane (the receiving magnetic loop and the relative signal processing unit will be described in detail in Part II of this paper).

Since the wires placed on the ground are much longer than the radius of the solenoid worn by the runner, to evaluate the *e.m.f.* they have been assumed as two straight parallel lines of infinite length.

Therefore, if the wire is fed by a sinusoidal current $I=I_{0}\cos(\omega t)$, and assuming the small loop approximation, the induced voltage amplitude is:

(1) $$V_{0}=\left|\frac{\partial \Phi_{B}}{\partial t}\right|=\frac{{\omega \mu }_{0}I_{0}{\mathrm{Nr}}^{2}}{2\sqrt{x^{2}+{h_{c}}^{2}}}\cos\theta$$

**Figure 3:**
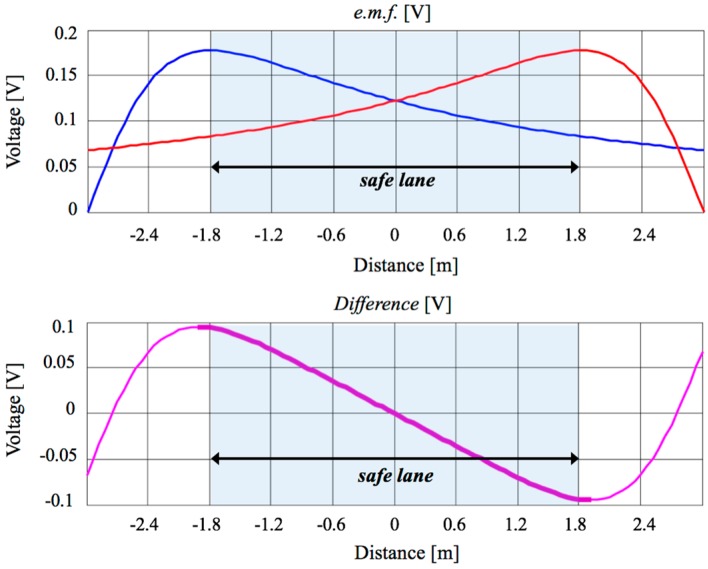
Top: theoretical evaluation of the induced electromotive force *e.m.f.*, according to (Equation (1)). The blue and red lines refer to the external and internal wire respectively. Bottom: difference of the received signals.

As the detecting coil (placed at a constant height *h_c_*) moves from *x* = 0 (i.e., above the wire) to a higher value, a variation of the induced voltage (Equation (1)) occurs, due to the change of the distance between the wire and loop and to the concatenation angle θ = arctan (*h_c_*/*x*). By repeating the same considerations for the other wire, Figure 3 top is obtained, where the two *e.m.f.* are depicted for the case of *h_c_* = 1 m, *I*_0_ = 1 A and *f* = 100 kHz. The presence of a region between the two wires where both signals are monotone, one is decreasing and the other is increasing, can be clearly seen. Their difference (Figure 3, bottom) highlights a monotone behavior in the central region. This zone of linearity has been chosen to delimit the safe lane and the intensity of the difference signal is used to guide the athlete, modulating the warning message according to his position in the lane.

As Figure 3 shows, at the center of the safe lane, the difference is zero (meaning that the two signals are equal) and, as long as the athlete maintains his position in the central region, no warning is produced. As he approaches one of the boundaries during running, the signal difference becomes greater or smaller than zero and a suitable warning is generated to help the athlete move to the left or right until the signal difference becomes zero again. It is worth noting that the choice of the work frequency is the result of a trade-off between two counter-posed aspects. The first requirement is the maximization of the induced voltage (Equation (1)), which entails the use of a frequency as high as possible. The second requirement is the uniformity of the current I_0_ along the whole cable, which leads to the use of a very low frequency. This condition is reasonably matched if the length of the lane is smaller than a quarter wavelength, therefore λ/4 > 400 m, i.e., *f* < 187.5 kHz in free space.

Moreover, in this frequency range, other EM conditions are fulfilled: radiation is very low and for system design, the magnetic field only can be considered, neglecting the contribution of the electric field [10].

As described in [11,12], the *f* = 100 kHz is one of the free frequencies allowed for a short range inductive device.

## 3. System Design: Transmitting Unit

In this section, the most important steps for the design of the generator feeding the wires are described. In particular, the current *I*_0_ has to be determined and generated, but this parameter strongly depends on the loading effect of the ground on the wire.

From an EM point of view, the structure is a current source lying at the interface between air, a lossless dielectric, and the ground, a dissipative medium. The rigorous approach to describe the EM fields in a stratified medium has been adopted, because it allows to account for the effects of the currents induced into the ground on the loop impedance value.

### 3.1. Loop Impedance Evaluation

The Green function of a stratified medium is analytically known [13], and this allows us to express the impedance of the ground loop in a closed form. Applying the Faraday law to the loop, we achieve the circuit representation of the structure:

(2) $$-V_{0}+I_{0}\frac{C}{\sigma 2\pi a\delta }=k_{0}^{2}\oint_{C}\bar{\bar{\Pi }}\left(\bar{I_{0}}\right)\cdot d\bar{l}$$

where

- C is the loop length
- Π is the Hertz’s Potential
- σ is the wire conductivity
- a is the wire radius
- δ is the penetration depth in the wire

In (2), the internal inductance of the wire is neglected because it is much smaller than the external inductance. Inserting in (2) the expression of Π, and defining the impedance as *Z_L_ = V*_0_*/I*_0_, we achieve

(3) $$Z_{L}=\frac{R}{\sigma a\delta }+\frac{{\omega \mu }_{0}R^{2}}{8\pi^{2}}\int_{0}^{2\pi }\int_{0}^{2\pi }[\int_{0}^{2\pi }\int_{0}^{\infty }\xi \;d\xi \;d\alpha \frac{2e^{-{j\gamma }_{1}a}}{\left(\gamma_{1}+\gamma_{2}\right)}\cdot e^{j\xi R\left[\cos\left(\alpha -\phi \right)-\cos\left(\alpha -\phi \prime \right)\right]}]\cos\left(\phi -\phi^{\prime }\right)d\phi \;d\phi^{\prime }$$

where:

(4) $$\gamma_{1}=\sqrt{k_{1}^{2}-\xi^{2}};\;\gamma_{2}=\sqrt{k_{2}^{2}-\xi^{2}}$$

In (3), the first term indicates the resistance of the cable, while the second term allows an evaluation of the cable impedance considering the presence of the lossy ground; *k*_1_ and *k*_2_ are wavenumbers for free space and ground respectively. In (3), the assumption of a circular track of radius R, with a total length equal to the one of a real stadium, has been adopted. This leads to a significant reduction of the computational effort, with negligible effect on the accuracy. For the evaluation of (3) the integral over the spectral variable *ξ* has been numerically computed up to a proper asymptotic value *ξ*_as_, and then the asymptotic part of the integral has been evaluated with the method of the stationary phase.

In order to validate the theoretical method, a comparison between calculated and measured values has been done considering a wire of length C = 400 m lying on common soil, whose electric properties have been chosen as *ε_r_* = 10 and *σ* = 1 S/m, according to [14,15]. The value *Z_L_* = 41 + j586 ω is obtained, which implies a value of inductance of about 900 μH. Actually, the value of *Z_L_* depends on the type of ground. Unfortunately, the values of the electric parameters of the ground are not precisely known, therefore it is difficult to carry out a comparison between corresponding theoretical and experimental situations. Table 1 reports the values of the impedance measured for the wire lying on three different typical surfaces. We can observe that in the case of soil, whose typical dielectric parameters were used to compute the theoretical value, the agreement is quite good, whereas in the other cases the variability of the unknown electric parameters affects the comparison. It is worth noting the value of the resistance, which highlights the need for a rigorous approach: if we consider only the wire ohmic losses, we obtain the value of 5 *ω*, about one order of magnitude smaller than the measured or calculated values. The great difference is due to the current density flowing into the lossy ground and induced by the magnetic field generated by the cables. The use of the accurate model (Equation (1)) allows us to account correctly for this aspect.

The knowledge of the impedance, and hence the load seen by the generator, allows us to optimize the system design. In particular, a mismatching between the generator and the load may cause dangerous power reflection for the electronic circuit, providing too low current such that the system cannot be used.

### 3.2. Signal Generator

**Figure 4:**
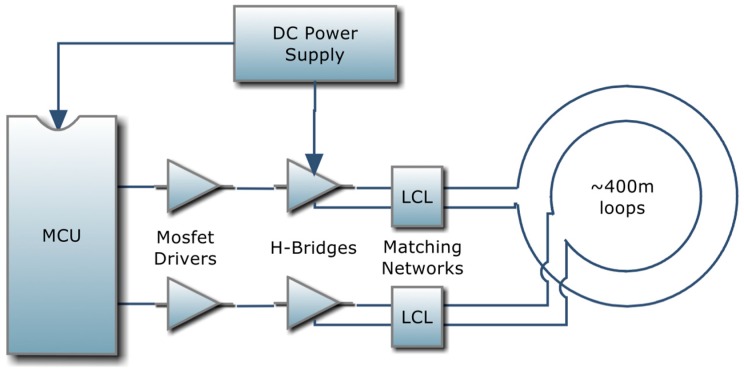
Schematic representation of the signal generation unit, where MCU and LCL respectively indicate the microcontroller unit and the inductances/capacitor matching network.

**Figure 5:**
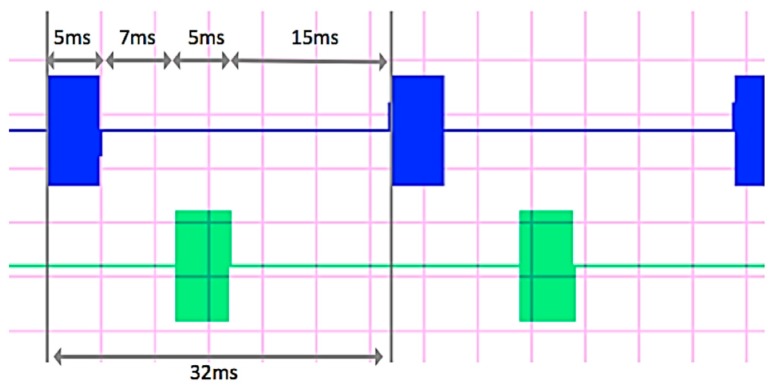
Current signals generated for the two wires.

This section accurately describes the generation of the signal, which consists of a sinusoidal carrier at the frequency *f* = 100 kHz, modulated in amplitude by a square wave. The model described in the previous section allowed to evaluate the need for a pulse with a peak to peak amplitude of, at least, 300 mA, in order to have a detectable *e.m.f*. Figure 4 depicts a schematic representation of the signal generation unit to obtain the current signal shown in Figure 5.

The signal is characterized by a pulse repetition time of 32 ms and a duration of 5 ms (blue trace in Figure 5). The same signal is delivered to the second wire after a delay time of 7 ms (green trace in Figure 5). This sequence defines different delay times between current pulses, so that each new pulse on the first wire is generated 15 ms after each pulse on the second wire is ended. This sequence is detected by the receiving unit through the generated magnetic field and allows to discriminate between signals coming from the two different wires.

**Figure 6:**
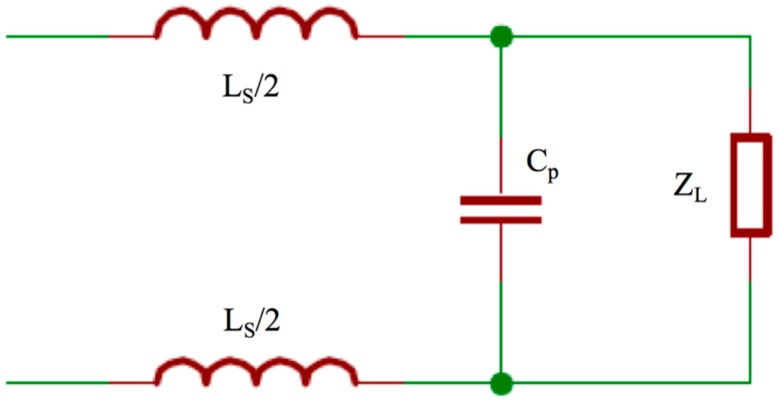
Matching network used to provide the load with higher power.

The theoretical value of the loop input impedance calculated through (1) highlights a problem posed to properly connect the 400 m long loop with the generator: the expected impedance value is high, therefore a suitable matching network has to be designed so that the desired current flows along the wire. The matching network of Figure 6 was adopted. The capacitance in parallel compensates the load inductance, whereas the series inductances provide the desired resistance value for the generator.

In order to design a suitable matching network, further measurements, Table 2, were carried out to evaluate the actual load conditions, also taking into account the coupling between the two cables.

Once the actual *Z_L_* is known, we chose a commercially available high power inductor, considering two main constraints: the *L_s_* value has to limit the current provided by the generator to a maximum of 15 A, and the corresponding stray resistance *R_s_* has to be low enough to reduce the internal ohmic losses. The *L_s_* chosen was 29 μH with a corresponding small *R_s_* of 0.92 *ω*. Finally, we calculated the suitable *C_p_* able to load the generator with a pure resistive impedance. Using the obtained values, the simulated current flowing on the wire, with a power supply of 12 Vdc, was 476 mA peak to peak, which satisfies the requirement of being higher than 300 mA.

### 3.3. Generator Realization

**Figure 7:**
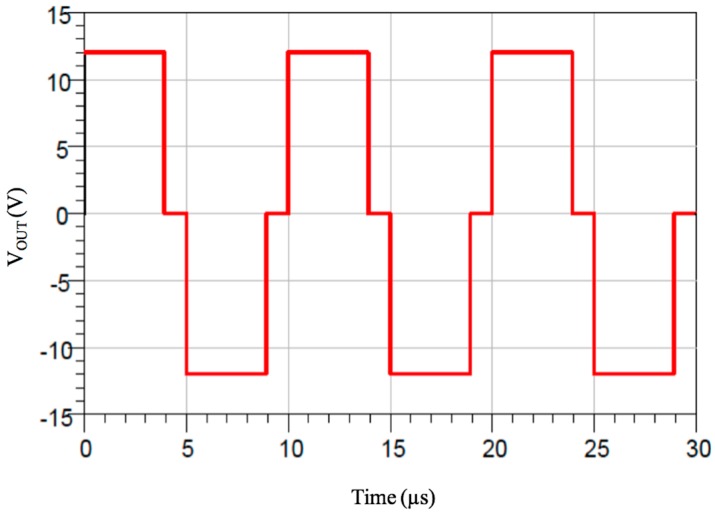
Modified square wave used to drive the H-Bridge.

The generation of a square wave at 100 kHz was implemented using a microcontroller unit (MCU). Since, for this function, very few resources (in terms of ROM, RAM and pin count) are needed, the chosen MCU is an Atmel AVR ATtiny44 (4096 bytes of flash memory, 320 bytes of RAM, 256 bytes of Eeprom). The final stage that injects current to the 400 m long loop has been designed as an H-bridge driver to allow both polarities to feed the loop, thus doubling the voltage/current drive, with respect to a single switch drive. Between the MCU pins and each bridge, some current drivers have been used to speed up the charge/discharge of the gates of the Mosfets and to ensure their full conduction. To prevent simultaneous conduction of the Mosfets of the same branch of the bridge, a little dead time has been added during transitions. Therefore, the resulting waveform is a modified square wave, and the voltage at the terminals of the generator is the one depicted in Figure 7 for the case of a supply of 12 Vdc.

The 100 kHz frequency is so obtained by feeding 12 V/0 V for 3.9375 μs, a dead time of 1.0625 μs (all mosfets off), then reversed output 0 V/12 V for 3.9375 μs, finally a dead time of 1.0625 μs again. The total period time is obviously 10 μs, whose accuracy is dependent on the 16 MHz crystal oscillator used, which has ±0.5% of frequency tolerance and ±0.3% of frequency stability. The measured current flowing on the wire is *I_pk-pk_* = 450 mA, slightly lower than the simulated one, mainly because of the measurement uncertainty of some components. The time-division solution simplifies the receiver hardware, despite a more complex software implementation. The most important feature of the adopted solution is the considerable reduction of power amount dissipated on the transmitter, which delivers only short bursts of 100 kHz wave to each channel, with a duty cycle of 15.6% for each wire (5 ms over a total period of 32 ms).

## 4. Discussion and Conclusions

In this paper, a fixed infrastructure designed to allow the autonomous running of blind athletes has been presented and the transmitting unit has been described in detail. It consists of a RF generator and two wires, placed directly on the ground perimeter of a standard athletics track. The currents flowing on the wires generate two magnetic fields, delimiting a virtual safe lane inside which a blind athlete can autonomously run without risks. A rigorous approach has been used to describe the EM fields in a stratified medium, allowing to account for the effects of the currents induced into the ground on the loop impedance value. The knowledge of the correct load seen by the generator has given us the possibility of optimizing the system design and in particular to introduce a suitable matching network so as to obtain the desired value for the currents and then for the magnetic fields.

The whole transmitting structure proposed in this paper has been realized with standard commercial components and therefore it is cheap and easy to install, both for fixed and for removable infrastructures. Moreover, the dimensions of the lane realized and the particular signals used, permit the use of the infrastructure by more than one athlete at the same time without interferences. In fact, the proposed device is suitable for use in stadiums, race tracks or indoor placement, while for races or training in an outdoor environment, for example the marathon on the road, the *Race-track system* presents an important shortcoming, since two long wires should be placed for the entire path with consequent operational issues. In this scope, innovative GPS-based devices (DGPS, Galileo and so on) might be integrated into the EM system design, mutually offsetting strengths and weaknesses: the proposed system would be ideal for stadiums or indoor placement, while the GPS technology would be more suitable for road racing. Anyway, starting from the theoretical result reported in Figure 3 (inside the safe invisible hallway, the difference signal has a linear behavior and a maximum *e.m.f.* value of about 100 mV over a width of 1.8 m) and considering a reasonable sensibility of 10 mV for the receiving system, it is possible to estimate an accuracy of about 20 cm, comparable with the accuracy of modern GPS technology. Nevertheless, it is worth noting that the running track system has been mainly designed to confine an athlete inside an invisible hallway rather than to localize him, as GPS devices are able to do.

To conclude, it is important to assess that the magnetic fields generated by the currents flowing into the wires are safe for the runners. The International Commission on Non-Ionizing Radiation Protection (ICNIRP) defines a magnetic field limit of 80 A/m for occupational people and 21 A/m for the general public [16]. During tests, a safety distance of 0.5 m was chosen, and with the maximum used current of 440 mA, peak-to-peak value, a magnetic field of about 0.05 A/m was measured, far below the exposure limit. Therefore, the system is not dangerous for the health of the users.

## Figures and Tables

**Table 1:** Measured values of Impedance and Inductance for different grounds.

Scenario	*Z* [Ω]	*L* [mH]
Soil	41.1 + j528	0.842
Tarmac	62 + j604	0.961
Tartan	151 + j740	1.178

**Table 2:** Measured values of Impedance of 400m wires

Cable Measured	*Z_L_* [Ω] at 100 kHz
Internal cable (without external cable)	151 + j740
Internal cable (open circuited external cable)	143 + j755
Internal cable (short circuited external cable)	125 + j747
External cable (without internal cable)	100 + j744
External cable (open circuited internal cable)	108 + j740
External cable (short circuited internal cable)	93.2 + j737
